# Polysplenia Syndrome Associated with Situs Inversus Abdominus and Type I Jejunal Atresia

**Published:** 2011-07-30

**Authors:** Farooq Rasool, Bilal Mirza

**Affiliations:** Department of Paediatric Surgery, The Children's Hospital and the Institute of Child Health Lahore, Pakistan; 1 Department of Paediatric Radiology, The Children's Hospital and the Institute of Child Health Lahore, Pakistan

**Dear Sir**

Polysplenia is a presence of two or more spleens in a patient and polysplenia syndrome refers to its association with various organ abnormalities in abdomen and chest. In about 20% cases of polysplenia syndrome situs inversus is present [1]. Polysplenia syndrome in association with situs inversus abdominus and intestinal atresia is rarely reported. We are presenting a case of polysplenia syndrome associated with situs inversus abdominus and type-I jejunal atresia.

A 2-day-old female baby was admitted with bilious vomiting and failure to pass meconium. There was a history of polyhydramnios in the mother. The weight of the baby was 2.5 kg with no significant perinatal problems. X-ray abdomen erect posture revealed three air fluid levels. A per rectal examination yielded mucous only. A preoperative diagnosis of intestinal atresia was made.

At laparotomy the stomach was found in the right side of the abdomen with seven spleens located behind and along greater curvature of the stomach (Fig. 1). Four spleens were of equal size. Three spleens were small and globular and one of these had torsion and was gangrenous. The liver was present on the left side. There was a type-I jejunal atresia about 20 cm distal to the dudeno-jejunal junction (Fig. 2). The gangrenous spleen was removed and an end to end jejuno-jejunal anastomosis performed. The post operative recovery was uneventful. Further investigations in the postoperative period showed levocardia and no structural cardiac anomaly. Patient remained well at follow up.

**Figure:**
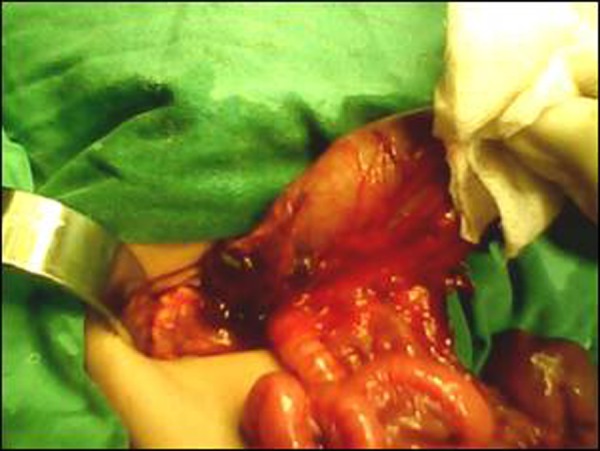
Figure 1: Showing right sided stomach and multiple spleens

**Figure:**
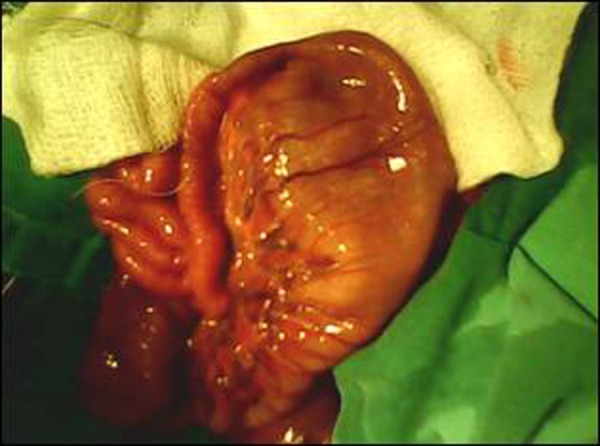
Figure 2: Type-I jejunal atresia in the same patient

The normal arrangement of thoracic and abdominal organs is called situs solitus. Situs ambiguous or heterotaxy is a congenital abnormal position of abdominal and thoracic viscera. Situs inversus refers to reversed or mirrored position of the abdominal and thoracic organs. In fewer cases Situs inversus involves only abdominal organs where it is called situs inversus abdominus (SIA) [2].

Polysplenia syndrome, as initially described by Helwig in 1929, is often recognized in the childhood though about 10% cases are not detected until adulthood. More than 40% cases of polysplenia syndrome have cardiac anomalies and majority of such children do not survive beyond 5th year of life.

They are mostly diagnosed incidentally during work up of other associated anomalies. Plain x-ray chest and abdomen can give clue about abnormal location of heart in chest and stomach gas shadow on the right side of abdomen. CT scan, MRI, angiography, echocardiography are important tools for ascertaining the location and number of spleens, location of other organs in the chest and abdomen, and identification of other associated anomalies [2, 3].

Polysplenia sometimes is diagnosed during abdominal surgery for some other reasons [2, 3]. The presentation in case of polysplenia syndrome depends upon the presence and severity of the associated anomalies. The common features are vague abdominal pain, nausea, and vomiting. The other features are specific to the associated anomalies. We believe that vague abdominal pain can be attributed to the episodes of partial torsion of the smaller globular spleens resulting in ischemia and thus pain. Nevertheless, the vague abdominal pain may also occur due to associated anomalies such as malrotation. 

The location and number of spleens are variable. They are often located in the right abdomen and along the greater curvature of stomach or behind it. Their number may range from 2 to 16. In our case there were seven spleens, located behind the stomach and one along greater curvature.

Small sized spleen with narrow pedicle can twist around its pedicle thus resulting in infarction as found in the index case. There is limited data about the clinical presentation in children with infarction of one of spleens in this syndrome. We have managed a patient with two spleens and retroperitoneal immature teratoma. In that patient one of the spleens was twisted and gangrenous at operation without any preoperative abdominal symptoms related to this pathology. This observation was also noted in the index case where the presentation was of neonatal intestinal obstruction.

Polysplenia syndrome and situs inversus are reported to be associated with heterotaxy of various organs, congenital heart defects, malrotation, biliary atresia, immotile cilia syndrome, annular and short pancreas, intestinal atresia, preduodenal portal vein, reverse rotation of the intestine, congenital lobar emphysema, vena cava abnormalities, and so on. Very few cases have described concomitant presence of polysplenia syndrome, situs inversus abdominus and intestinal atresia. Situs inversus abdominus usually presents with polysplenia, but, in very few cases asplenia can be found which is further associated with immunocompromized state thus resulting in poor prognosis. Abdur-Rahman et al reported a case of situs inversus abdominus, asplenia and reverse rotation of intestine [1, 3]. In our case the heterotaxy was present in the abdomen with seven spleens and type-I jejunal atresia which is a very rare combination though reported in literature.

## Footnotes

**Source of Support:** Nil

**Conflict of Interest:** None declared
